# Ultrasound-guided minimally invasive autopsy as a tool for rapid post-mortem diagnosis in the 2018 Sao Paulo yellow fever epidemic: Correlation with conventional autopsy

**DOI:** 10.1371/journal.pntd.0007625

**Published:** 2019-07-22

**Authors:** Amaro Nunes Duarte-Neto, Renata Aparecida de Almeida Monteiro, Janaina Johnsson, Marielton dos Passos Cunha, Shahab Zaki Pour, Amanda Cartagenes Saraiva, Yeh-Li Ho, Luiz Fernando Ferraz da Silva, Thais Mauad, Paolo Marinho de Andrade Zanotto, Paulo Hilário Nascimento Saldiva, Ilka Regina Souza de Oliveira, Marisa Dolhnikoff

**Affiliations:** 1 Departamento de Patologia, Faculdade de Medicina da Universidade de São Paulo, São Paulo, Brasil; 2 Departamento de Diagnóstico por Imagem, Universidade Federal de São Paulo, São Paulo, Brazil; 3 Laboratório de Evolução Molecular e Bioinformática (LEMB), Departamento de Microbiologia, Instituto de Ciências Biomédicas II, Universidade de São Paulo, São Paulo, Brasil; 4 Divisão de Moléstias Infecciosas e Parasitárias, Hospital das Clínicas da Faculdade de Medicina da Universidade de São Paulo, São Paulo, Brasil; 5 Departamento de Radiologia e Oncologia, Faculdade de Medicina da Universidade de São Paulo, São Paulo, Brasil; University of Iowa, UNITED STATES

## Abstract

**Background:**

New strategies for collecting post-mortem tissue are necessary, particularly in areas with emerging infections. Minimally invasive autopsy (MIA) has been proposed as an alternative to conventional autopsy (CA), with promising results. Previous studies using MIA addressed the cause of death in adults and children in developing countries. However, none of these studies was conducted in areas with an undergoing infectious disease epidemic. We have recently experienced an epidemic of yellow fever (YF) in Brazil. Aiming to provide new information on low-cost post-mortem techniques that could be applied in regions at risk for infectious outbreaks, we tested the efficacy of ultrasound-guided MIA (MIA-US) in the diagnosis of patients who died during the epidemic.

**Methodology/principal findings:**

In this observational study, we performed MIA-US in 20 patients with suspected or confirmed YF and compared the results with those obtained in subsequent CAs. Ultrasound-guided biopsies were used for tissue sampling of liver, kidneys, lungs, spleen, and heart. Liver samples from MIA-US and CA were submitted for RT-PCR and immunohistochemistry for detection of YF virus antigen. Of the 20 patients, 17 had YF diagnosis confirmed after autopsy by histopathological and molecular analysis. There was 100% agreement between MIA-US and CA in determining the cause of death (panlobular hepatitis with hepatic failure) and main disease (yellow fever). Further, MIA-US obtained samples with good quality for molecular studies and for the assessment of the systemic involvement of the disease. Main extrahepatic findings were pulmonary hemorrhage, pneumonia, acute tubular necrosis, and glomerulonephritis. One patient was a 24-year-old, 27-week pregnant woman; MIA-US assessed the placenta and provided adequate placental tissue for analysis.

**Conclusions:**

MIA-US is a reliable tool for rapid post-mortem diagnosis of yellow fever and can be used as an alternative to conventional autopsy in regions at risk for hemorrhagic fever outbreaks with limited resources to perform complete diagnostic autopsy.

## Author summary

Reliable mortality information is of paramount importance to establish sound public health policies. Autopsy is an important tool not only for determining the cause of death, but also for the detection of novel diseases. In the last decades, we have been globally identifying an unprecedented number of emerging infections. Therefore, there is great interest in the development of less invasive and low-cost tools for the accurate post-mortem diagnosis in fatal cases. Minimally invasive autopsy (MIA), conceived as targeting diagnostic biopsies of key organs by needle puncture, has been proposed as an alternative to conventional autopsy (CA) for the determination of cause of death in developing countries. In this research, we tested the efficacy of MIA in the post-mortem diagnosis of 20 patients with suspected or confirmed yellow fever who died during the recent epidemic of yellow fever that occurred in Brazil. There was a perfect agreement between MIA and CA in determining the cause of death (hepatic failure) and main disease (yellow fever) in all patients with confirmed yellow fever. This finding indicates that MIA can be used as an alternative to CA in regions at risk for infectious disease outbreaks with limited resources to perform conventional autopsies.

## Introduction

In the last decades, we have been globally identifying an unprecedented number of emerging infections [1]. As a result, considerable effort is currently being made by several actors from governmental and non-governmental institutions for better preparedness for the next expected emerging threats to public health worldwide [2–4]. Therefore, there is great interest in the development of tools for the early detection of infectious outbreaks for the establishment of appropriate measures [2,5].

Autopsy has been an important and indisputable diagnostic tool for the detection of novel diseases. In the past years, our group has performed autopsy studies to describe new aspects of human pathology of emerging infectious diseases, such as measles, leptospirosis, and influenza A(H1N1)pdm09 [6–8]. However, the global distribution of facilities capable of performing the procedure is markedly uneven. New strategies for collecting post-mortem tissue samples are necessary, particularly in areas where outbreaks of infectious diseases are occurring and where the identification of the causative agent as well as its effects on target organs is a public health priority [4,5,9].

Minimally invasive autopsy (MIA) has been proposed as an alternative to conventional autopsy, conceived as targeting small diagnostic biopsies by needle puncture of key organs, with or without the guidance of any imaging technique. The use of computed tomography (CT) and CT-angiography, associated with needle biopsy, has been proposed as feasible for diagnosis of common causes of death [10,11]. However, these techniques can only be performed in centers of excellence, requiring substantial budgets. More recently, ultrasound-guided tissue sampling (MIA-US) has also been tested, with promising results [12–18]. This methodology represents a portable, rapid and low-cost post-mortem technique, which may be especially useful in countries where mortality data are largely unavailable [2,13–18].

Viral hemorrhagic fevers (VHFs) are severe viral infections that may present as hemorrhagic disease with fatal multi-organ failure. In endemic areas, they can cause long-lasting epidemics with great impact on human morbidity and mortality. Yellow fever (YF), in particular, is a re-emerging disease, endemic in tropical regions of South America and sub-Saharan Africa. It is a mosquito-borne flavivirus-induced VHF, with a high case-fatality rate, clinically manifested as hepatic dysfunction, renal failure, coagulopathy, and shock [19]. The investigation of deaths related to VHFs should put MIA-US into perspective among other potential diagnostic strategies.

From the end of 2017 to mid-May 2018, we experienced a YF epidemic in the southern region of Brazil. During this period, 498 autochthonous confirmed YF cases were registered in the state of Sao Paulo, with 176 deaths (fatality rate: 35.4%). A substantial part of the fatalities (80 cases) was referred to the autopsy service at Sao Paulo University Medical School. Aiming to provide new information on low-cost post-mortem techniques that could be applied in at-risk regions in Brazil as well as worldwide, we tested the efficacy of MIA-US in the post-mortem diagnosis of 20 patients who died with suspected or confirmed YF during the recent epidemic in Brazil.

## Methods

### Ethics statement

This prospective observational study was approved by the University of Sao Paulo School of Medicine Internal Review Board (CAAE protocol number: 18781813.2.0000.0068). Informed written consent was obtained from the next of kin.

From January 23, 2018 to February 27, 2018, 20 deceased patients with suspected or confirmed YF underwent MIA using ultrasound-guided percutaneous core needle biopsy. Conventional autopsy (CA) was performed subsequently by a distinct group of pathologists, blinded to the MIA-US results.

**Fig 1:**
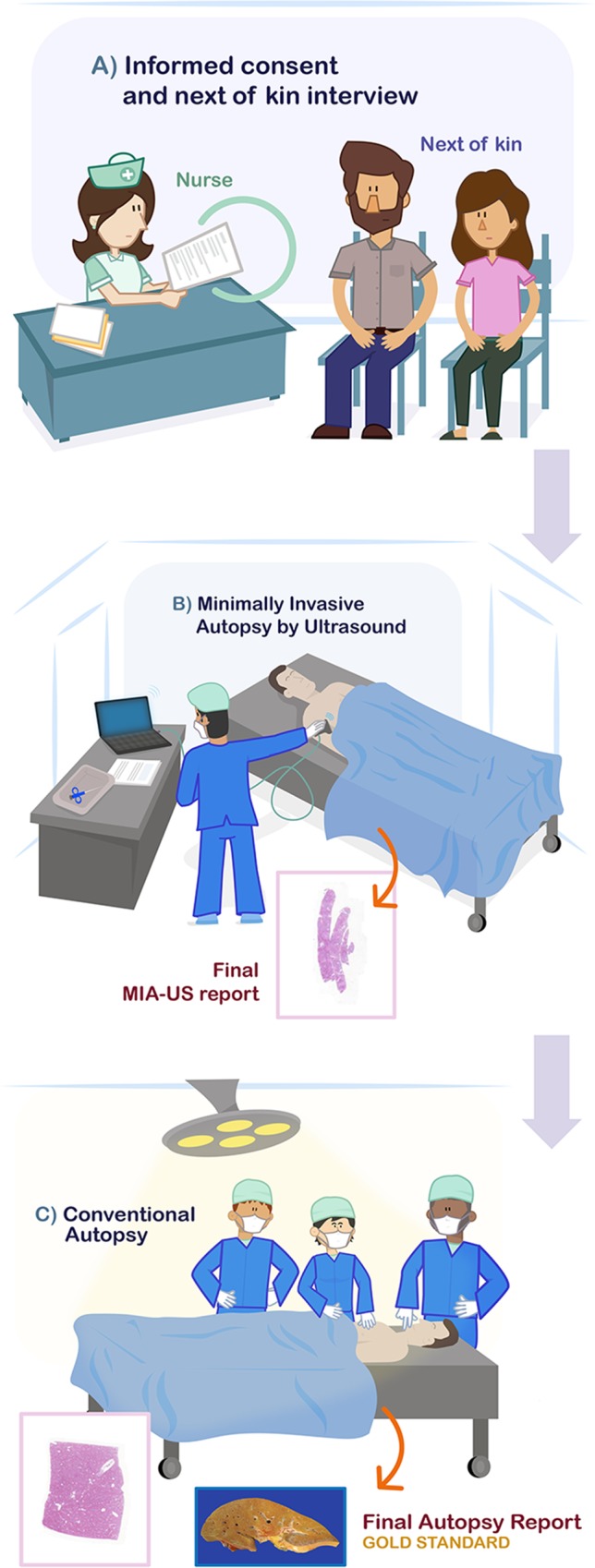
Representation of the procedures performed at the autopsy room. (A) Interview with next of kin. (B) MIA-US procedure. Insert shows an example of histological slide of liver biopsy. (C) Conventional autopsy performed right after MIA-US by pathologists unaware of the MIA-US findings. Insert shows an example of histological slide of liver at autopsy.

Fig 1 illustrates the sequence of the autopsy procedures.

All the patients included in the study fulfilled the following criteria: 1) patients died with suspected or confirmed yellow fever; 2) an autopsy was requested by the clinician; 3) death occurred during business hours, when interventional radiologists were available to perform the MIA-US; 4) informed consent to perform the autopsy was given by a family member.

The case definition of YF employed during the epidemic period was established by the Brazilian Ministry of Health and the Health Department of the State of Sao Paulo. Suspected cases referred to those patients who had a sudden onset of high fever associated with jaundice and/or hemorrhages, who lived or had visited areas with cases of YF, YF epizootics in non-human primates, or isolation of yellow fever virus (YFV) in vectors, regardless of the vaccine status for YF, during the preceding 15 days. Confirmed cases referred to those patients who had compatible clinical presentation and laboratory confirmation by at least one of the following methods: positive serum IgM (MAC-ELISA) (performed in 10 of 20 patients); detection of YFV-RNA by RT-PCR in blood samples (performed in 17 of 20 patients); and histopathology compatible for YF hepatitis with detectable YF antigen in tissues by immunohistochemistry (wild, SP strain, hyperimmune, IAL–SP, Brazil) [20]. In all of the cases, viral hepatitis (A, B, and C) and dengue fever were excluded by serology and/or RT-PCR.

### MIA-US protocol

The procedures were performed at the “Image Platform in the Autopsy Room”, a research center in the University of Sao Paulo Medical School, located next to the Autopsy Service of Sao Paulo University (https://pisa.hc.fm.usp.br/). Radiologists and pathologists were aware of the information provided by the next of kin prior to autopsy.

All US examinations were performed by interventional radiology physicians, who were trained to perform gray-scale post-mortem US during a period of 8 weeks. In total, fifteen exams were necessary in order to achieve thorough ultrasound imaging and good quality biopsy samples. We used a SonoSite M-Turbo portable ultrasound (Fujifilm, Bothell, WA, USA) with broadband and multifrequency transducers: C60x (5–2 MHz Curved) and HFL38X (13–6 MHz Linear) and DICOM medical images.

Tissue samples from the liver (at least two samples), lungs (three samples from each lung), both kidneys (one sample each), spleen (one sample), and heart (one sample) were collected under ultrasound guidance using a large core (14-gauge) needle biopsy. A final MIA-US diagnosis was provided by conjunct analysis of US and biopsy data, blinded to CA results.

### Conventional autopsy protocol

The autopsies were performed following the Letulle technique, where all the organs are removed *en masse*, requiring dissection of each organ [21]. Tissue samples were collected from all body systems. Both biopsy and autopsy samples were submitted for routine histological examination, and stained with hematoxylin-eosin (H&E). Brown-Brenn, Ziehl-Neelsen, and Grocott stains were used to detect bacteria, acid-fast bacilli, and fungi, respectively, when required.

### RT-PCR protocol

Eleven liver biopsy samples and all autopsy liver samples were submitted for reverse transcriptase-polymerase chain reaction (RT-PCR) for detection of YFV-RNA. Liver samples measuring 1 cm^3^ were stored at −70°C. The tissue was macerated, the nucleic acid extraction was performed using the TRIzol reagent (Life Technologies, Carlsbad, CA, USA), and carried out according to the manufacturer’s instructions. Molecular detection of YFV was performed using the AgPath-ID One-Step RT-PCR Reagents (Ambion, Austin, TX, USA) with specific primers/probe previously described [22]. To identify cases of adverse vaccine response (i.e., fatal cases associated with the vaccine virus) we used primers/probe specific for the vaccine virus [23]. qRT-PCR reactions consisted of a step of reverse transcription at 45°C for 10 min, enzyme activation at 95°C for 10 min, and 40 cycles at 95°C for 15 s and 60°C for 45 s for hybridization and extension with the use of ABI7500 equipment (Thermo Fisher Scientific, Waltham, MA, USA).

### Immunohistochemistry (IHC) protocol

All biopsy and autopsy liver samples were submitted for IHC for detection of YFV antigens as previously described [24]. Briefly, after antigen retrieval using Tris-EDTA at pH 9.0, the sections were incubated overnight with the primary antibody (goat anti-human IgM polyclonal anti-arbovirus, provided by Institut Pasteur de Dakar, Dakar, Senegal, 1:20,000) at 4°C [25]. The slides were incubated with biotinylated secondary antibody (Reveal–Biotin-Free Polyvalent HRP DAB, Spring Bioscience, cod. SPD-125) and chromogen (Dako Liquid DAB+ Substrate Cromogen System, Dako, cod. K3460) and counterstained with Harris-hematoxylin (Merck, Darmstadt, Germany). The primary antibody was tested in liver samples from patients with virus hepatitis (A, B, and C), herpes virus, cytomegalovirus, adenovirus, dengue virus, *Treponema pallidum*, *Leptospira*, and atypical mycobacteria infections, obtained from our autopsy archive. The IHC reaction resulted negative in all these samples.

### Comparison between MIA-US and conventional autopsy data

We compared the MIA-US diagnosis with the CA diagnosis of the cause of death and main disease. In addition, we performed a descriptive analysis of the concordances and discrepancies between the diagnoses of MIA-US and CA in all organs analyzed.

## Results

All patients who died during the yellow fever epidemic that fulfilled the inclusion criteria were included in the study (n = 20).

Of the 20 patients who died with suspected (n = 11) or confirmed YF (n = 9), 17 had their diagnosis confirmed after autopsy by histopathological and molecular analysis. A total of nine in ten patients had positive serology. Serum YFV-RT-PCR was performed in 17 patients, and 16 patients showed positive results. However, eight patients died before their serum RT-PCR results were obtained, and their diagnoses were established after autopsy.

YF was not confirmed in three patients, as described below. Interestingly, MIA-US could determine the main disease and cause of death in these three patients.

First we present the results of the 17 patients with confirmed YF.

### Clinical data

The patients comprised 13 men and 4 women, with a median age of 47 (24–86) years. Fourteen patients died at our institution and three were referred for autopsy. Sixteen patients lived or traveled to the epizootic areas of the city. One patient lived in urban area and had YF vaccine-associated viscerotropic disease (YEL-AVD), which was confirmed by liver RT-PCR. Six patients received YF vaccination 0–3 days before death; only one of them presented with YEL-AVD. One patient was a 24-year-old, 27-week pregnant woman. The median timespan between the occurrence of symptoms and hospital admission was 4 days (1–15) and that between the occurrence of symptoms and death was 8 days (6–21). The most frequent associated clinical conditions were alcoholism (n = 7), smoking (n = 5), and systemic arterial hypertension (n = 4). One patient had undergone liver transplantation three days before death for hepatic failure due to YF. The primary symptoms upon hospital admission were fever, jaundice, myalgia, anorexia, abdominal pain and hemorrhagic phenomena. The main alterations in the laboratory data were related to acute liver failure, as well as renal dysfunction and metabolic acidosis.

Table 1 presents detailed clinical data of the 17 patients.

**Table 1:** Epidemiological, clinical and laboratorial characteristics of 17 patients with confirmed yellow fever.

Characteristic	Total (n = 17)
**Epidemiological data**	
**Age, median (range), y**	47 (24–86)
**Male sex, No. (%)**	13 (76.5)
**Previous YFV-vaccination, n (%)**	6 (35.3)
**Days before symptoms (range)**	2 days (0–3)
**Exposure to epizootics areas, n (%)**	16 (94)
**Diagnostic criteria for YF, n (%)**	
**Clinical criteria**	17 (100)
**Epidemiological criteria**	17 (100)
**Serology (IgM) (n = 10)**	9 (90)
**Serum YFV-RT-PCR positivity**	16 (94)

**Abbreviations:** YFV, yellow fever virus; YF, yellow fever; RT-PCR, reverse transcriptase polymerase chain reaction.

### MIA-US

Adequate biopsy samples from the liver (100%), kidneys (94%), and lungs (88%) were obtained in majority of patients. More limited samples were obtained from the spleen (82%) and heart (76%).

Liver biopsies showed panlobular hepatitis with severe steatosis and midzonal apoptotic bodies, which were characteristic of fatal YF, in all samples analyzed (100%). All liver samples were submitted for IHC and 11 liver samples were submitted for RT-PCR for detection of YFV. IHC revealed positive results in 16 samples (94%), and RT-PCR revealed positive results in all 11 samples (100%).

Lung samples showed pulmonary involvement (73%) that included alveolar hemorrhage (67%) and pneumonia (60%). Pulmonary aspergillosis was confirmed in a single lung biopsy. Kidney alterations included acute tubular necrosis (100%) and a mesangial proliferative glomerulonephritis (63%). Spleen biopsies showed lymphoid hypoplasia (93%), splenitis (86%), and hemophagocytosis (43%). Heart biopsies showed interstitial edema (85%) and fiber hypertrophy (54%). In the pregnant woman, MIA-US could be used to assess the placenta and provided adequate placental tissue for histological analysis.

### Conventional autopsy

The cause of death in the 17 patients was hepatic failure. Associated organ-specific hemorrhages and/or hemorrhagic shock was present in 16 patients. One patient presented with septic shock.

All patients presented with panlobular hepatitis with severe steatosis and midzonal apoptotic bodies. IHC was positive in 15 (88%) of 17 liver samples, and RT-PCR was positive in 17 (100%) liver samples. One patient had undergone liver transplantation after developing YF-induced hepatic failure and the graft was infected. One patient presented with YEL-AVD, confirmed by liver and spleen RT-PCR. Other autopsy liver findings were hepatomegaly, ischemic centrilobular necrosis, and alcoholic cirrhosis (two cases). Ascites was detected in 10 (59%) patients. Table 2 presents detailed liver findings at MIA-US and CA for the 17 patients.

**Table 2:** Post-mortem liver findings in minimally invasive-ultrasound guided autopsy (MIA-US) and conventional autopsy of 17 patients with confirmed yellow fever.

Pathological liver findings	MIA-US	Conventional autopsy
**Adequate tissue sampling**	17 (100)	17 (100)
**Hepatomegaly**	8 (47)	12 (71)
Typical YF-hepatitis*	17 (100)	17 (100)
**Centrilobular ischemic necrosis**	4 (24)	4 (24)
**Portal fibrosis**	1 (6)	-
**Alcoholic cirrhosis**	-	2 (12)
**Positive results on IHC**	16 (94)	15 (88)
**Positive results on RT-PCR**	11/11 (100)	17 (100)

*Typical YF-hepatitis: panlobular hepatitis with severe steatosis and midzonal apoptotic bodies; IHC: immunohistochemistry for Yellow Fever virus antigen; RT-PCR: reverse transcriptase-polymerase chain reaction for yellow fever virus RNA.

At the respiratory system, alveolar hemorrhage (94%) and pneumonia (53%) were the main pulmonary autopsy findings. Pleural effusion (41%), diffuse alveolar damage (41%), and bronchoaspiration (18%) were also observed. Two patients presented with fungal pneumonia (one aspergillosis and one mucormycosis) and associated pulmonary necrosis secondary to mycotic thrombus. Two patients presented with pulmonary embolism. The main kidney alterations were acute tubular necrosis (94%) and mesangial proliferative glomerulonephritis (88%). Other kidney findings were interstitial fibrosis, nephrosclerosis, hypertensive nephropathy, vascular thrombosis, and pyelonephritis. Spleen findings included lymphoid hypoplasia (100%), splenitis (94%) and cytophagocytosis (50%). One patient underwent splenectomy. The primary cardiac changes at autopsy included fiber hypertrophy (76%), interstitial edema (71%), myocardiosclerosis (65%), and coronary atherosclerosis (65%).

Autopsy also showed organ alterations that were not assessed by MIA-US, such as pancreatic changes (ischemic pancreatic changes, peripancreatic steatonecrosis, and alcoholic pancreatic interstitial fibrosis), central nervous system (CNS) changes (cerebral edema, perivascular hemorrhage, and cerebral herniation), gastrointestinal (GI) bleeding, skin perivascular inflammation, lymphoid hypoplasia in lymph nodes, and bone marrow with erythroid depletion and hemophagocytosis.

### CA and MIA-US findings in three patients in whom the diagnosis of yellow fever was not confirmed

One patient was a 19-year-old man who died due to perforated appendicitis and sepsis. Purulent fluid leaked from the abdominal cavity when the radiologist performed US-guided liver and kidney biopsies, indicating an intra-abdominal infection. Lung biopsy in this case showed secondary acute lung injury with intense pulmonary hemorrhage. One patient was an 87-year-old woman with sepsis due to bacterial pneumonia and pyelonephritis, both observed respectively on lung and kidney US-guided biopsies. The third patient was a 14-year-old boy with acute myeloid leukemia. Liver and kidney samples from MIA-US showed infiltration of atypical myeloid CD34 positive cells (blasts).

### Concordances and discrepancies between MIA-US and CA in 17 FA patients

#### Main disease and cause of death

MIA-US and CA determined the cause of death (hepatic failure) and main disease (YF) in all 17 (100%) patients.

#### Liver

Panlobular hepatitis was observed in all MIA-US and CA liver samples. Liver fibrosis/cirrhosis was present in two patients at CA and was not observed in the biopsies.

#### Lungs

Alveolar hemorrhage and pneumonia were the main pulmonary findings both at CA and MIA-US. Alveolar hemorrhage was present in 16 (94%) of 17 autopsies and in 10 (67%) of 15 lung biopsies. MIA-US was able to detect pneumonia in all nine patients, including the patient with aspergillosis. Mucorales infection (one case) and pulmonary embolism (two cases) were not observed at lung biopsy. The lung samples in two patients were not successfully obtained using MIA-US. Both of these patients presented with alveolar hemorrhage, and one presented with pulmonary embolism at CA.

#### Kidneys

Acute tubular necrosis (94%) and mesangial proliferative glomerulonephritis (88%) were the main kidney alterations observed at CA. Acute tubular necrosis was observed in all MIA-US kidney samples (16/16) and mesangial proliferative glomerulonephritis in 63%. Hypertensive nephropathy was present at CA in four patients; three of them were identified by kidney biopsy. Interestingly, in one patient with pyelonephritis, the etiologic agent (*Aspergillus* sp) was identified at MIA-US but not at CA.

#### Spleen

Lymphoid hypoplasia, splenitis, and cytophagocytosis were the main findings on both CA and MIA-US.

#### Heart

Interstitial edema and fiber hypertrophy were the main heart changes observed at CA and MIA-US. Myocardiosclerosis and myocarditis, observed at CA in 11 and 3 patients, respectively, were not detected by heart biopsies.

**Fig 2:**
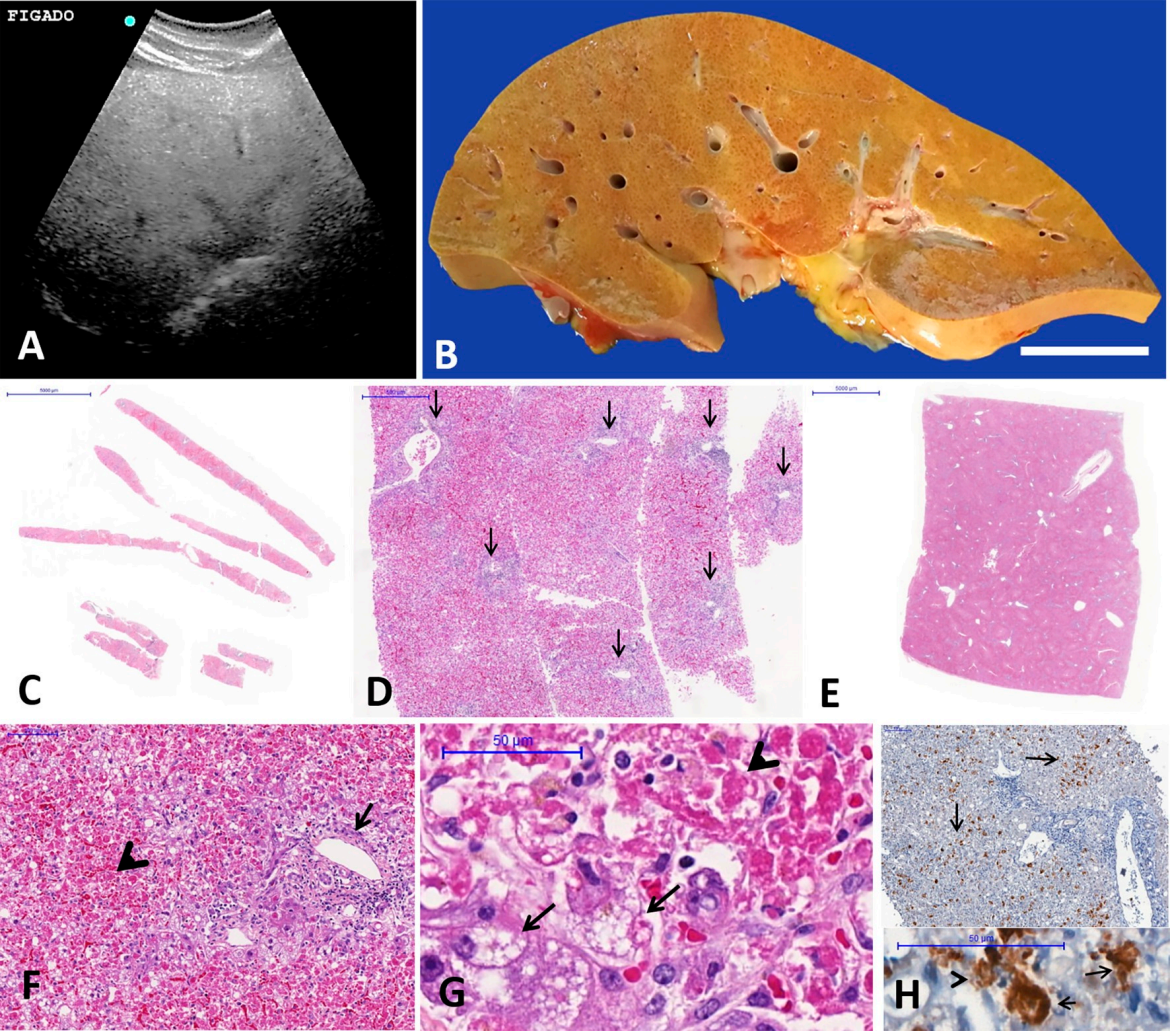
Post-mortem liver examination from fulminant yellow fever cases performed by ultrasound-guided minimally invasive autopsy (MIA-US) and conventional autopsy (CA). (A) Post-mortem abdominal ultrasound demonstrating hepatic steatosis; (B) gross exam of the liver with YF hepatitis, showing yellowish color due to diffuse steatosis; (C–D) adequate post-mortem liver samples, collected through MIA-US (C), with sufficient lobular areas and number of portal tracts (arrows in D); (E) micrograph of an H&E slide at low magnification demonstrating the usual sampling of the hepatic parenchyma collected at CA; (F) micrograph of YF hepatitis–portal tract with mild lympho-mononuclear inflammatory infiltrate (arrow) and severely damaged mid-zonal hepatocytes (arrowhead); (G) micrograph showing mid-zonal hepatocytes with steatosis (arrows) and acidophilic degeneration–the so-called Councilman-Rocha-Lima bodies (arrowhead), Kupffer cell hyperplasia, and minimal inflammatory reaction; (H) micrographs showing detection of YFV antigen in the liver parenchyma by immunohistochemistry, mainly in the mid-zonal area in a sample collected by MIA-US (arrows). At the bottom, Councilman-Rocha-Lima bodies (arrows) and Kupffer cells (arrowhead) labeled with anti-YFV (IHC). C–G: H&E staining, H: immunohistochemistry for YFV.

**Fig 3:**
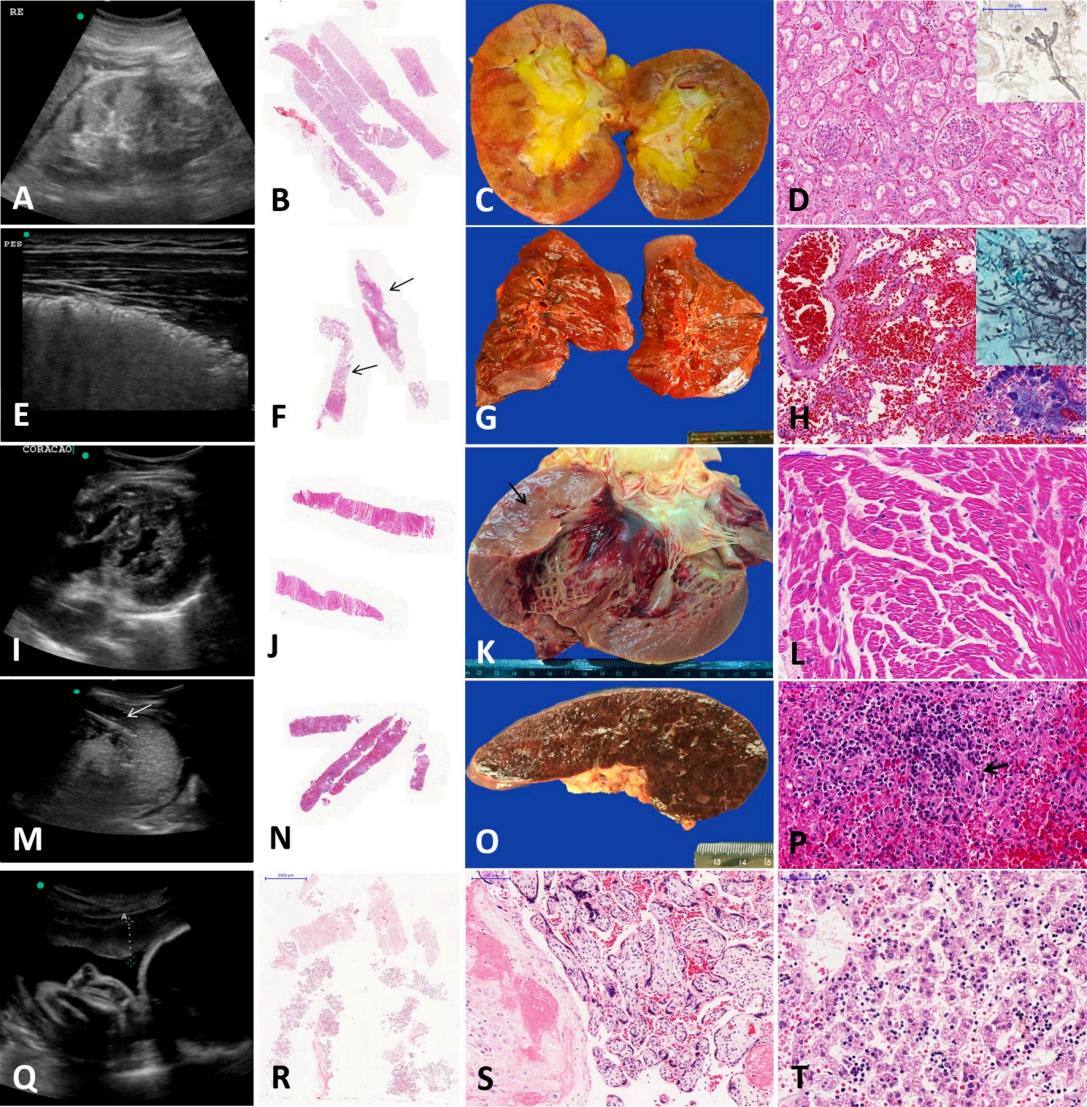
*Post-mortem* examination of fulminant yellow fever cases performed by ultrasound-guided minimally invasive autopsy (MIA-US) and conventional autopsy (CA). (A-D) *Kidneys*: nephromegaly detected by US (A); H&E slide, at low magnification, showing kidney samples collected by MIA-US (B); gross examination of kidneys showing edema and icterus (C); microscopy showing mesangial cell hyperplasia and acute tubular necrosis (D). The inset shows typical *Aspergillus* hyphae invading an interstitial arterial vessel in the renal parenchyma, diagnosed in the left kidney from one case, only in the MIA-US sample (reticulin stain). (E-H) *Lungs*: lung hepatization detected by US (E); H&E slide, at low magnification, showing lung samples collected by MIA-US with foci of pneumonia (arrows) (F); gross exam of lungs with red hepatization and edema (G); microscopy of lungs (H) showing alveolar hemorrhage (HE, left), invasive pulmonary aspergillosis (Grocott stain, right top) and pneumonia with colonies of Gram-positive cocci (H&E, right bottom). (I-L) *Heart*: a concentrically hypertrophic left ventricle detected by US (I); H&E slide, at low magnification, showing heart samples collected by MIA-US (J); gross exam of a heart exhibiting left ventricle hypertrophy (arrow) and subendocardial hemorrhages (K); microscopy showing hypertrophic cardiomyocytes and interstitial edema (L). (M–P) *Spleen*: an image of MIA-US at the moment of needle insertion in splenic mass (arrow) to collect post-mortem samples (M); H&E slide, at low magnification, showing spleen samples collected by MIA-US (N); gross exam of the spleen showing diffuse congestion on the cut surface (O); microscopy revealing splenitis, hemorrhage, and lymphoid hypoplasia (P). (Q-T) *MIA-Us of a pregnant woman*: the US image shows the uterus, placenta, amniotic fluid and fetus (Q); H&E slide, at low magnification, showing the placental samples collected by MIA-US (R); histology of placenta with maternal decidua and villi (S); the fetal liver sampled by MIA-US showing ischemic damage and extramedullary hematopoiesis (T).

Figs 2 and 3 illustrate the MIA-US and CA main findings in the liver and other organs, respectively.

## Discussion

The present results demonstrate the effectiveness of MIA-US in the post-mortem diagnosis of the main disease (YF) and cause of death (hepatic failure) in a group of patients evaluated during the epidemic of YF that occurred in early February 2018 in Sao Paulo, Brazil. We found a 100% concordance between MIA-US and CA for the diagnosis of the main disease and cause of death, which validated our initial proposition that this diagnostic method could be a rapid and viable alternative to CA in determining post-mortem diagnosis of a VHS.

The excellent correlation between MIA-US and CA in this group of patients may be explained by some factors. First, the acknowledged accuracy of ultrasound to guide needles into the viscera affected by this viral disease, especially the liver, kidneys, and lungs. Second, important technical aspects were applied to obtain tissue samples, involving the use of the portable ultrasound equipment and Tru-Cut 14G needles. Third, procedures were performed by interventional radiologists after a training period. The ultrasound guidance, ensuring the researchers about the organ or region that underwent needle biopsies, favored good quality samples in most patients, thus allowing precise histopathologic diagnosis to be made. Technical limitations were observed in the access to the spleen and heart of some patients, due to their anatomical locations, i.e. the heart being partially obscured by the sternum and the spleen by the rib cage and intestinal gas distension. The procedure turned out to be a simple and reliable method of post-mortem tissue sampling that could be applied in remote areas after adequate training.

Our results are in concert with those of other researchers [12–18,26,27]. In the series by Fariña et al. [12], there was an 83% diagnostic concordance between MIA-US and CA. Castilho et al. [14] reported a success rate of 83% for the determination of cause of death by MIA-US in a study of 30 patients with a predominance of infectious diseases. Recently, Martinez et al. [15] compared MIA-US and CA in 30 patients and obtained an 89.5% concordance for the detection of an infectious agent directly involved in the cause of death. Conversely, Cox et al. [13] found a 57% concordance between MIA-US and CA for infectious major diagnoses, mainly present in the lungs (63%), liver (44%), and spleen (34%). Result differences among studies possibly relate to different patient populations and different causal mixes.

Our results further showed that MIA-US is also a reliable tool to obtain samples with sufficient quality for molecular studies and microorganism identification and to assess the systemic involvement of the disease. Besides typical hepatic changes observed on histological examination, IHC and/or RT-PCR allowed definite viral detection in 94% of the patients. These results encourage further development of local technologies in endemic regions, which might provide access to real-time research data, improving epidemic preparedness.

In addition to the liver, the main affected organs at CA were the lungs and kidneys [19]. Lung biopsies showed that pulmonary hemorrhage and suppurative pneumonia were important complications of YF infection, significantly contributing to death. Besides the more prevalent bacterial pneumonia, MIA-US was able to detect severe pulmonary aspergillosis in one patient, demonstrating an immunosuppressive state associated with fatal YF. Shock-induced acute tubular necrosis was related to kidney dysfunction and could be detected in all kidney biopsies. Interestingly, in one patient with pyelonephritis, the etiologic agent (*Aspergillus* spp.) could be identified at MIA-US but not at CA. YF-induced glomerulonephritis could be observed in most kidney biopsies [24]. One of the 17 patients was a 24-year-old, 27-week pregnant woman. In this specific case, MIA-US was able to assess the placenta and provided adequate placental tissue for analysis. Therefore, we believe that MIA-US can be an important tool to study maternal deaths and maternal-fetal transmission.

The limitations of MIA-US in the present study were related to changes observed at autopsy that were not assessed by MIA-US. These mainly included changes related to GI tract and CNS. Shock-induced ischemic pancreatic alterations, steatonecrosis, and gastrointestinal bleeding, as well as cerebral edema, were common and important findings that certainly contributed to death. Therefore, for diseases that primarily affect the CNS and/or GI tract, strategies to include assessment of these organs should be considered.

In the past years, we have been globally identifying an unprecedented number of emerging infections [1,4,5,18]. This phenomenon is probably associated with a complex interaction of factors such as human behavior, environmental changes and vector proliferation worldwide [1]. Precise post-mortem diagnosis during the first periods of an emerging epidemic would represent an improvement in identifying the specific etiologic agent, with substantial impact in disease monitoring. For instance, it is quite plausible that if post-mortem brain sampling had been performed in the early cases of microcephaly in Northeastern Brazil, the identification of Zika virus infection could have been made earlier [28]. Moreover, protective measures could have been taken more effectively. However, such measures are hindered by the lack of autopsies in areas prone to be affected by emerging infectious agents. In addition, because of its minimal invasiveness, MIA-US may represent a safer procedure to health authorities who investigate the emerging infectious diseases of high (Ebola, for instance) or unknown lethality.

In this scenario, our results indicate that MIA-US represents a portable, low-cost post-mortem technique that can be useful for the rapid monitoring and surveillance of infectious diseases spread and outbreaks. The adoption of consolidate protocols, associated with international partnerships to use the resources of molecular diagnosis to regions where such resources are scarce, may, theoretically, expand the horizons of surveillance of global infectious threatens.

In conclusion, our results show that MIA-US is a reliable tool for the post-mortem diagnosis of YF and can be used as an alternative to conventional autopsy in regions at risk for viral hemorrhagic fever of different etiologies.

## Supporting information

S1 Checklist STROBE checklist. (DOC)

## References

[1] Fauci AS, Morens DM. The perpetual challenge of infectious diseases. N Engl J Med. 2012;366: 454–61. 10.1056/NEJMra1108296 22296079

[2] Bassat Q, Ordi J, Vila J, Ismail MR, Carrilho C, Lacerda M, et al Development of a post–mortem procedure to reduce the uncertainty regarding causes of death in developing countries. Lancet Glob Health. 2013;1: e125–126. 10.1016/S2214-109X(13)70037-8 25104253

[3] Massad E, Coutinho FA, Wilder–Smith A. Is Zika a substantial risk for visitors to the Rio de Janeiro Olympic Games? Lancet. 2016;388: 25. 10.1016/S0140-6736(16)30842-X 27323918

[4] Perkins MD, Dye C, Balasegaram M, Bréchot C, Mombouli JV, Røttingen JA, et al Diagnostic preparedness for infectious disease outbreaks. Lancet. 2017;390: 2211–2214. 10.1016/S0140-6736(17)31224-2 28577861

[5] Pigott DM, Deshpande A, Letourneau I, Morozoff C, Reiner RC Jr, Kraemer MUG, et al Local, national, and regional viral haemorrhagic fever pandemic potential in Africa: a multistage analysis. Lancet. 2017;390: 2662–2672. 10.1016/S0140-6736(17)32092-5 29031848 PMC5735217

[6] Vargas PA, Bernardi FD, Alves VA, Gianotti MA, Almeida OP, Saldiva PH, et al Uncommon histopathological findings in fatal measles infection: pancreatitis, sialoadenitis and thyroiditis. Histopathology. 2000;37: 141–146. 10931237 10.1046/j.1365-2559.2000.00951.x

[7] Del Carlo Bernardi F, Ctenas B, da Silva LF, Nicodemo AC, Saldiva PH, Dolhnikoff M, et al Immune receptors and adhesion molecules in human pulmonary leptospirosis. Hum Pathol. 2012;43: 1601–1610. 10.1016/j.humpath.2011.11.017 22436623

[8] Mauad T, Hajjar LA, Callegari GD, da Silva LF, Schout D, Galas FR, et al Lung pathology in fatal novel human influenza A (H1N1) infection. Am J Respir Crit Care Med. 2010;181: 72–79. 10.1164/rccm.200909-1420OC 19875682

[9] Gates B, Gates M. Bill and Melinda Gates: Autopsies Could Prevent Epidemics, Save Countless Lives. Available at: https://www.huffpostbrasil.com/entry/autopsies-on-children-bill-melinda-gates_n_7289610 [Accessed 12 Oct. 2018].

[10] Blokker BM, Wagensveld IM, Weustink AC, Oosterhuis JW, Hunink MG. Non–invasive or minimally invasive autopsy compared to conventional autopsy of suspected natural deaths in adults: a systematic review. Eur Radiol. 2016;26: 1159–1179. 10.1007/s00330-015-3908-8 26210206 PMC4778156

[11] Bolliger SA, Filograna L, Spendlove D, Thali MJ, Dirnhofer S, Ross S. Postmortem imaging-guided biopsy as an adjuvant to minimally invasive autopsy with CT and postmortem angiography: a feasibility study. AJR Am J Roentgenol. 2010;195: 1051–1056. 10.2214/AJR.10.4600 20966306

[12] Fariña J, Millana C, Fdez-Aceñero MJ, Furió V, Aragoncillo P, Martín VG, et al Ultrasonographic autopsy (echopsy): a new autopsy technique. Virchows Arch. 2002;440: 635–639. 10.1007/s00428-002-0607-z 12070604

[13] Cox JA, Lukande RL, Kalungi S, Van Marck E, Van de Vijver K, Kambugu A, et al Needle autopsy to establish the cause of death in HIV-infected hospitalized adults in Uganda: a comparison to complete autopsy. J Acquir Immune Defic Syndr. 2014;67: 169–176. 10.1097/QAI.0000000000000290 25072614

[14] Castillo P, Ussene E, Ismail MR, Jordao D, Lovane L, Carrilho C, et al Pathological Methods Applied to the Investigation of Causes of Death in Developing Countries: Minimally Invasive Autopsy Approach. PLoS One. 2015;10: e0132057 10.1371/journal.pone.0132057 26126191 PMC4488344

[15] Martínez MJ, Massora S, Mandomando I, Ussene E, Jordao D, Lovane L, et al Infectious cause of death determination using minimally invasive autopsies in developing countries. Diagn Microbiol Infect Dis. 2016;84: 80–86. 10.1016/j.diagmicrobio.2015.10.002 26508103

[16] Castillo P, Martínez MJ, Ussene E, Jordao D, Lovane L, Ismail MR, et al Validity of a Minimally Invasive Autopsy for Cause of Death Determination in Adults in Mozambique: An Observational Study. PLoS Med. 2016;13: e1002171 10.1371/journal.pmed.1002171 27875530 PMC5119723

[17] Castillo P, Hurtado JC, Martínez MJ, Jordao D, Lovane L, Ismail MR, et al Validity of a minimally invasive autopsy for cause of death determination in maternal deaths in Mozambique: An observational study. PLoS Med. 2017;14: e1002431 10.1371/journal.pmed.1002431 29117196 PMC5695595

[18] Bassat Q, Castillo P, Martínez MJ, Jordao D, Lovane L, Hurtado JC, et al Validity of a minimally invasive autopsy tool for cause of death determination in pediatric deaths in Mozambique: An observational study. PLoS Med. 2017;14: e1002317 10.1371/journal.pmed.1002317 28632739 PMC5478091

[19] Monath TP, Vasconcelos PF. Yellow fever. J Clin Virol. 2015;64: 160–173. 10.1016/j.jcv.2014.08.030 25453327

[20] Mascheretti M, Tengan CH, Sato HK, Suzuki A, de Souza RP, Maeda M, et al Yellow fever: reemerging in the state of Sao Paulo, Brazil, 2009. Rev Saude Publica 2013;47: 881–889. 10.1590/s0034-8910.2013047004341 24626492

[21] Hanzlick RL. The autopsy lexicon: suggested headings for the autopsy report. Arch Pathol Lab Med. 2000;124: 594–603. 10.1043/0003-9985(2000)124<0594:TAL>2.0.CO;2 10747319

[22] Diallo D, Sall AA, Diagne CT, Faye O, Hanley KA, Buenemann M, et al Patterns of a sylvatic yellow fever virus amplification in southeastern Senegal, 2010. Am J Trop Med Hyg. 2014;90: 1003–1013. 10.4269/ajtmh.13-0404 24615140 PMC4047721

[23] Avelino-Silva VI, Leal FE, Sabino EC, Nishiya AS, da Silva Freire M, Blumm F, et al Yellow fever vaccine viremia following ablative BM suppression in AML. Bone Marrow Transplant. 2013;48: 1008–1009. 10.1038/bmt.2012.277 23334273

[24] De Brito T, Siqueira SA, Santos RT, Nassar ES, Coimbra TL, Alves VA. Human fatal yellow fever. Immunohistochemical detection of viral antigens in the liver, kidney and heart. Pathol Res Pract. 1992;188: 177–181. 10.1016/S0344-0338(11)81176-3 1594489

[25] Mengesha Tsegaye M, Beyene B, Ayele W, Abebe A, Tareke I, Sall A, et al Sero–prevalence of yellow fever and related Flavi viruses in Ethiopia: a public health perspective. BMC Public Health. 2018;18: 1011 10.1186/s12889-018-5726-9 30107830 PMC6092792

[26] Cox JA, Lukande RL, Lucas S, Nelson AM, Van Marck E, Colebunders R. Autopsy causes of death in HIV-positive individuals in sub-Saharan Africa and correlation with clinical diagnoses. AIDS Rev. 2010;12: 183–194. 21179183

[27] Karat AS, Tlali M, Fielding KL, Charalambous S, Chihota VN, Churchyard GJ, et al Measuring mortality due to HIV–associated tuberculosis among adults in South Africa: Comparing verbal autopsy, minimally–invasive autopsy, and research data. PLoS One. 2017;12: e0174097 10.1371/journal.pone.0174097 28334030 PMC5363862

[28] Martines RB, Bhatnagar J, de Oliveira Ramos AM, Davi HP, Iglezias SD, Kanamura CT, et al Pathology of congenital Zika syndrome in Brazil: a case series. Lancet. 2016;388: 898–904. 10.1016/S0140-6736(16)30883-2 27372395

